# Cardiac Infiltration as the First Manifestation of Acute Lymphoblastic Leukemia: A Systematic Review

**DOI:** 10.3389/fonc.2022.805981

**Published:** 2022-01-27

**Authors:** Zhi Luo, Jun Cheng, Yanggan Wang

**Affiliations:** ^1^ Department of Internal Medicine, Zhongnan Hospital of Wuhan University, Wuhan, China; ^2^ Medical Research Institute of Wuhan University, Wuhan, China

**Keywords:** acute lymphoblastic leukemia, cardiac problems, management, recommendations, misdiagnosis and mistreatment

## Abstract

Cardiac symptoms or signs as the first manifestations in acute lymphoblastic leukemia patients are sporadically reported which lead to misdiagnosis or delayed diagnosis due to lack of clinical experience and improper diagnosis procedures. Here, we documented the clinical features, procedures of diagnosis, treatments, and outcomes from the so-far reported 30 lymphoblastic leukemia cases that initially presented as cardiac problems and provided management recommendations based on the experiences and lessons learned from these patients to help physicians avoid misdiagnosis and improper treatment.

## 1 Introduction

Leukemic cardiac infiltration is frequently observed in postmortem autopsies (1) with incidence rate of 30%–44% (2–4). However, 99% of patients with cardiac infiltration were asymptomatic. Notably, a large sample of autopsy study (2) reported that cardiac infiltrations (51/116) were microscopic and occurred in the late stage of acute leukemia. As a result, the definite antemortem diagnosis of leukemic cardiac infiltration was rare (5, 6). In addition, the incidence of leukemic cardiac infiltration was significantly higher in acute myeloid leukemia than acute lymphoblastic leukemia (ALL) (1, 2). Therefore, macroscopic cardiac infiltration as the first manifestation of ALL is rather rare. In the real world, these patients usually first visit cardiologists, instead of oncologists. As a result, these patients usually missed the best treatment timeline due to the misdiagnosis or delayed diagnosis. Here, 30 scarce cases presented cardiac tamponade (7–14), cardiac mass (15–22), myocardium hypertrophy (23–30), or acute myocardial infarction (AMI) (31–36) as the first sign of ALL were carefully reviewed, and we provided recommendations of management procedures for the diagnosis and treatment in these atypical ALL patients.

## 2 Methods

A comprehensive literature search was performed using PubMed and the Cochrane database for English-language studies published from December 1985 through March 31, 2021. The following keywords were used: (“acute lymphoblastic leukemia” or “ALL”) and (“cardiac manifestation”, “cardiac disease”, “cardiac symptoms”, or “cardiac signs”). Additionally, the reference lists of all eligible cases were manually retrieved to obtain more literatures. Eventually, 30 cases that presented massive pericardial effusion or cardiac tamponade, cardiac mass, myocardium hypertrophy, or AMI as the first manifestation of ALL were included. Since this article is a review but not a clinical study, ethical approval is not applicable.

## 3 Results

In general, ALL patients with cardiac disorders as the first clinical manifestation showed a strong predominance in adolescent and middle-aged population. The most common cardiac abnormalities include persistent pericardial effusion or cardiac tamponade, severe cardiac hypertrophy, cardiac mass, and AMI-like manifestations.

### 3.1 Cardiac Tamponade as the First Manifestation of ALL

All of the eight reported T-cell acute lymphoblastic leukemia (T-ALL) cases presented massive pericardial effusion or cardiac tamponade as the first manifestation (**Table 1**). An adolescent predominance was observed with an average age of 19.8 years old (ranging from 7 to 45). Progressive dyspnea, cough, chest pain, and fever were recognized as the prominent symptoms, and typical signs include paradoxical pulse, elevated jugular venous pressure, and muffled heart sounds. In most cases, the resting heart rate was greater than 120 beats/min with a blood pressure usually lower than 100/60 mmHg. ECG was usually characterized as sinus tachycardia with low voltage. Notably, the initial blood test could be completely normal. The abnormal leukocyte associated with lymphoblast was observed at follow-up.

**Table 1:** Cardiac tamponade as first manifestation of acute lymphoblastic leukemia.

Age/gender/type/first manifestation/ reference	Initial symptoms/signs	Initial vital signs	Initial ECG	Initial blood test	Initial imaging examination	Definite diagnosis	Treatment regimen	Outcome
18/F/T-ALL/cardiac tamponade/ (7)	Dyspnea, cough, fever, pericardial pain, and pulsus paradoxus	PR: 160 beats/min BP: 70/60 mmHg	Low voltage	Normal	CR: (−) Chest CT: lymph nodal enlargement TTE: pericardial effusion and a large mediastinal mass	Peripheral blood smear: (−) Pericardiocentesis cytologic examination: (−) Lymph node biopsy: ALL BMB: ALL, 70% infiltration of lymphoid blasts Bone marrow immunophenotypic analysis: T-ALL	Pericardiocentesis + systemic chemotherapy	Symptoms: improved after pericardiocentesis Pericardial effusion: disappeared after 1 month of chemotherapy Survival: Yes, complete remission after chemotherapy
27/M/T-ALL/massive pericardial effusion/ (8)	Breathlessness, fever, ascites, pulsus paradoxus, high jugular venous pressure, and muffled heart sounds	BP: 120/80 mmHg	Low voltage	WBC: 2.7 × 10^9^/L Hb: 41 g/L PLT: 41 × 109 × 109/L	CR: pleural and pericardial effusion TTE: massive pericardial effusion with cardiac tamponade	Peripheral blood smear: 46% abnormal lymphocytes Pericardiocentesis cytologic examination: cells appeared frankly atypical, with scanty cytoplasm and lymphoid nuclear chromatin BMA: 90% lymphoblasts with periodic acid–Schiff (+) BMA: T-ALL blasts CD3(+)	Pericardiocentesis + systemic chemotherapy	Symptoms: improved after pericardiocentesis Pericardial effusion: initially reduced by chemotherapy but reaccumulated shortly. Survival: No, died of cardiorespiratory arrest on the 5th day of chemotherapy
45/F/T-ALL/cardiac amponade/ (9)	Breathlessness, cough, fever, retrosternal chest pain, pulsus paradoxus, high jugular venous pressure, and muffled heart sounds	PR: 120 beats/min BP: 100/60 mmHg T: 37.8°C	Low voltage and sinus tachycardia	WBC: 7.3 × 10^9^/L Hb: 73 g/L PLT: 45 × 10^9^/L	CR: globular cardiac silhouette and right pleural effusion TTE: large pericardial effusion with gross cardiac oscillation and diastolic collapse of right ventricle and atrium	Peripheral blood smear: 79% blasts BMA: T-ALL	Systemic chemotherapy	Symptoms: improved after chemotherapy Pericardial effusion: disappeared after 6 days of chemotherapy Survival: yes, complete remission 1.5 months later after chemotherapy
7/F/T-ALL/massive pericardial effusion/ (10)	Progressive dyspnea, cough, high jugular venous pressure, and muffled heart sounds	PR: 120 beats/min BP: 90/50 mmHg	ST segment depression and sinus tachycardia	WBC: 4.12 × 10^9^/L Hb: 91 g/L PLT: 268 × 10^9^/L	CR: cardiomegaly TTE: large pericardial effusion	Peripheral blood film: (−) Pericardiocentesis cytologic examination: (−) BMA: ALL-L2	Pericardiocentesis + systemic chemotherapy	Symptoms: improved after pericardiocentesis Pericardial effusion: disappeared after pericardiocentesis and chemotherapy Survival: No, died 1 year later during the third chemotherapy
15/F/T-ALL/massive pericardial effusion/ (11)	Progressive dyspnea, cough, chest pain, pulsus paradoxus, high jugular venous pressure, and diminished heart sounds	PR: 120 beats/min BP: 80/60 mmHg T: 36.5°C RR: 30 breaths/min	Low voltage and sinus tachycardia	WBC: 19.1 × 10^9^/L Hb: 145 g/L PLT: 202 × 10^9^/L	CR: bilateral pleural effusions and an enlarged cardiac silhouette, suspicious to wide mediastinum TTE:large pericardial effusion with diastolic collapse of the right atrium and ventricle	Peripheral blood smear: 40%lymphoblast Pericardiocentesis: cytologic examination: (−) Pericardial biopsy: (−) BMB: ALL, 90% blasts Bone marrow immunophenotypic: T-ALL-L2 CD3(+), CD7(+); CD10(+), CD19(+), CD20(−)	Pericardiocentesis + systemic chemotherapy	Symptoms: improved after pericardiocentesis Pericardial effusion: disappeared after pericardiocentesis Survival: yes, complete remission after chemotherapy
7/M/T-ALL/massive pericardial effusion/ (12)	Cough, chest pain, and diminish heart sounds	−	Sinus tachycardia	WBC: 10.7 × 10^9^/L Hb: 108 g/L PLT: 4.11 × 10^9^/L	CR: global cardiomegaly with mediastinal enlargement TTE: massive pericardial effusion with obvious compression of right cardiac chambers	Peripheral blood film: (−) Pericardiocentesis cytologic examination: large amount of blasts Pathological, cytogenetic, immunophenotypic, and immunohistochemical: T-ALL	Pericardiocentesis + systemic chemotherapy	Symptoms: improved after pericardiocentesis Pericardial effusion: disappeared within 2 weeks after chemotherapy Survival: unknown
25/M/T-ALL/cardiac tamponade/ (13)	Progressive dyspnea, cough, high jugular venous pressure, and muffled heart sounds	PR: 120 beats/min BP: 96/5 8mmHg RR: 28 breaths/min	Low voltage, and sinus tachycardia	Normal	CR: complete opacification of left lung due massive pleural effusion Chest CT: massive left pleural effusion, massive pericardial effusion, and a anterosuperior mediastinum mass TTE: massive pericardial effusion and a swinging heart	BMB: (−) Lumbar puncture biopsy: (−) Immunohistochemical: T-ALL, CD99(+), TdT(+) Flow cytometric analysis pericardial fluid: T-ALL	Pericardiocentesis + systemic chemotherapy	Symptoms: improved after pericardiocentesis Pericardial effusion: disappeared after pericardiocentesis and chemotherapy Survival: unknown
15/M/T-ALL/massive pericardial effusion/ (14)	Dyspnea, cough, chest pain, fever, fatigue, and palpitation	PR: 128 beats/min RR: 26 breaths/min	Sinus tachycardia	WBC: 38.1 × 10^9^/L Hb: 142 g/L PLT: 1,591 × 10^9^/L	CR: cardiomegaly Chest CT: pericardial (28 mm) and pleural effusions (20 mm), thickening of the intra-atrial septum and pericardium TTE: pericardial effusion, and thickened pericardium with hyperechogenity	Peripheral blood smear: 80% lymphoblast Pericardiocentesis cytologic examination: diffuse atypical lymphocyte infiltration around mesothelial cells BMB: 91% FAB L2 type lymphoblasts Flow cytometric analysis: CD4 64.8%, CD5 98.3%, CD7 99.5%, and CD2 35.7%.	Pericardiocentesis + chemotherapy	Symptoms: improved after pericardiocentesis Pericardial effusion: disappeared after 1 month of chemotherapy Survival: yes, complete remission after chemotherapy

T-ALL, T-cell acute lymphoblastic leukemia; F, female; M, male; PR, pulse rate; BP, blood pressure; RR, respiration rate; WBC, white blood cell; Hb, hemoglobin; PLT, blood platelet; CR, chest radiography; CT, computerized tomography; TTE, transthoracic echocardiography; BMB, bone marrow biopsy; BMA, bone marrow aspiration.

In imaging examinations (**Table 1**), chest radiography was used to screen pleural effusion, and chest computerized tomography (CT) was sensitive to lymph nodal or mediastinum mass detection. However, transthoracic echocardiography (TTE) was commonly used to detect pericardial effusion or cardiac tamponade, characterized by a swinging or oscillation heart with a diastolic collapse of the right ventricle and atrium. The present studies showed that chest CT scan had a positive rate of 25 (2/8) in detecting mediastinum involvement (**Table 1**).

T-ALL diagnosis with atypical manifestations often requires comprehensive tests, such as pericardiocentesis cytologic examination, peripheral blood smear, bone marrow aspiration (BMA), bone marrow biopsy (BMB), immunophenotypes, immunohistochemistry, and flow cytometric analysis. While BMA or BMB were usually used to make a definite diagnosis, immunophenotypic, immunohistochemical, and flow cytometric analyses were usually used as a tool of qualitative assessment (T-cell markers). The present studies showed that BMA and BMB had a higher positive rate (6/7, 85.7%) than peripheral blood smear (4/6, 66.7%) and pericardiocentesis (4/7, 57.1%) test (**Table 1**).

Once diagnosed with T-ALL, pericardiocentesis and systemic chemotherapy were adopted (**Table 1**). The tamponade symptoms were released significantly after pericardiocentesis. Five patients achieved a resolution of pericardial effusion after 6 days to 4 weeks of chemotherapy. Three patients achieved complete remission from ALL after 4 to 6 weeks of chemotherapy. A particular case obtained complete remission after 3 years of intermittent chemotherapy due to recurrent supraventricular tachycardia caused by chemotherapy; one patient died of cardiorespiratory arrest on the 5th day of chemotherapy, and one patient died of chemotherapy resistance after 1-year treatment. The prognosis of the other two male patients was unknown due to lost follow-up.

### 3.2 Cardiac Mass as the First Manifestation of ALL

Eight B-cell acute lymphoblastic leukemia (B-ALL) patients presented cardiac mass as the first manifestation (**Table 2**). Similar to the T-ALL, an adolescent demographic feature was observed with an average age of 17 years old (ranging from 10 to 38). Cardiac mass was usually single and located in the cardiac cavity. The incidence of cardiac mass in the left and right heart chambers was 37.5% (3/8) and 62.5% (5/8), respectively. The most common symptoms include progressive dyspnea, chest pain, and syncope, usually companied with hypotension (<120/80 mmHg) and fast heart rate (>100 beats/min). In addition, the right heart mass usually presented peripheral edema, while the left heart mass usually presented severe chest pain with inverted T wave and/or depressed ST segment in the surface ECG and slightly elevated cardiac troponin I (cTnI) levels (0.9–2.19 ng/ml). In most cases, remarkably elevated peripheral blood leukocytes were observed.

**Table 2:** Cardiac mass as first manifestation of acute lymphoblastic leukemia.

Age/gender/type/first manifestation/reference	Initial symptoms/signs	Initial vital signs	Initial ECG	Initial blood test	Initial imaging examination	Definite diagnosis	Treatment regimen	Outcome
17/M/Pre-B-ALL/right atrium mass/ (15)	Chest pain and shortness of breath	PR: 88 beats/min BP: 112/68 mmHg	I, aVL, V4–6 with inverted T waves	WBC: 6.96 × 10^9^/L Hb: 146 g/L PLT: 228 × 10^9^/L	Chest CT: right atrium and bilateral kidneys masses TTE: right atrium hypoechoic mass (5.1 × 6.8 cm) and thickened ventricular wall, displayed a hypokinesis and focal asynergy, LVEF: 61% PET-CT (18F-FDG): a strong tracer accumulation in right atrium and bilateral kidneys	BMB: (−) Kidney biopsy: diffuse proliferation of medium-sized cells with irregular nuclei Immunohistochemical: CD10(+), CD79a(+), MIC2(+), and TdT(+); CD3(−), UCHL-1(−), CD56(−), and CD34(−)	Chemotherapy + APBSCT	Symptoms: chest pain and dyspnea disappeared Cardiac mass: right atrium mass completely disappeared, and thickened LV wall was normalized after 70 days of chemotherapy LVEF: improved Survival: yes, complete remission after chemotherapy
15/M/Pre-B-ALL/right ventricle mass/ (16)	Fever, lethargy, headache, cough, papilledema, and hepatosplenomegaly	–	–	WBC: 63 × 10^9^/L Hb: 57 g/L PLT: 10 × 10^9^/L	CR: (−) TTE: a large echodense mass (3X5 cm) involving the anterior and lateral walls of the right ventricle CMR: right ventricular mass, isointense to cardiac muscle with signal characteristics different from thrombotic lesion	BMB: (+) Immunophenotyping: pre-B-ALL, CD2(+), T-cell related antigen(+), CD13(+), TdT(+), CD10(+), CD19(+), cytoplasmic u(+), surface Ig(−), CD20(−), HLA DR(+), CD34(+), CD13(+), CD3(−), CD7(−), CD33(−), MPO(−) Molecular analysis: a monoclonal B-cell population was detected in IgH gene	Chemotherapy	Symptoms: improved after chemotherapy Cardiac mass: right ventricular mass had completely disappeared after 5 days of chemotherapy Survival: yes, complete remission after chemotherapy
13/M/Pre-B-ALL/left ventricular mass/ (17)	Severe chest pain, fever, fatigue, loss of strength, and weight loss	PR: 125 beats/min BP: 100/65 mmHg RR: 18 beats/min	Sinus tachycardia and ST segment depression	WBC: 23.4 × 10^9^/L With 71% eosinophils Hb: 114 g/L PLT: 47 × 10^9^/L cTnI: 0.9 ng/ml	CR: pulmonary edema TTE: left ventricular apex (5 × 5 cm) hypermobile mass, LVEF (50%)	BMB: hyperplastic marrow, increased number of eosinophils, and 40% lymphoid blasts Flow cytometric analysis: CD10(+), CD19(+), CD20(+), and CD22(+); CD3(−), CD5(−), CD7(−), CD13(−), CD33(−), and CD34(−)	Chemotherapy	Symptoms: chest pain persisted Cardiac mass: The initial mobile ventricular mass had shrunk after 13 days of chemotherapy. LVEF: reduced from 35% to 10% after chemotherapy Survival: no, died of cardio respiratory failure on the 25th day of chemotherapy
17/F/Pre-B-ALL/left ventricular mass/ (18)	Chest tightness and dyspnea	–	–	WBC: 49.8 × 10^9^/L with 63% eosinophils cTnI: 2.19 ng/ml	Chest CT: diffuse mural thrombus that occurred more in association with the posterior wall TTE: intracardiac thrombus attached to the mitral valve at the left ventricular posterior wall	BMB: pre-B-ALL with 40% lymphoblasts and an increase of eosinophils Cytogenetic: t(3;6)(p22;p24) FIP1L1-PDGFRA fusion Gene (−)	Chemotherapy	Symptoms: improved after chemotherapy Cardiac mass: left ventricular endocardial thrombosis had completely disappeared Eosinophils: return to normal Survival: yes, complete remission 2 months later after chemotherapy
38/M/Pre-B-ALL/right ventricle and right atrium mass/ (19)	Peripheral edema and ascites	–	–	WBC: 14 × 10^9^/L with 51% lymphoblasts Hb: 132 g/L PLT: 73 × 10^9^/L NT-proBNP: 3,667 pg/ml	PET-CT (18F-FDG): intense tracer uptake of the cardiac lesion highly consistent with aggressive malignancy CMR: a hypermetabolic mass extending from the right ventricle to the atrium; pleural and pericardial effusions	Bone marrow and cardiac mass biopsy (inserts depict hematoxylin and eosin stain): B-ALL, paired box (PAX) 5(+), CD34(+), and terminal deoxynucleotidyl transferase(+) CD3(−), CD20(−), CD10(−), CD1a(−)	Chemotherapy	Unknown
77/M/B-ALL/left atrial mass/ (20)	Progressive dyspnea, dysphagia, odynophagia, and fevers	PR: 100 beats/min BP: 93/32 mmHg	Normal sinus rhythm with occasional premature ventricular complexes	WBC: 7.3 × 10^9^/L Hb: 92 g/L PLT: 267 × 10^9^/L	CR: a moderate increase in cardiac size and mild pulmonary edema Chest CT: a mediastinal mass with paratracheal lymphadenopathy TTE: an extensive left atrial tissue density mass CMR: a large mediastinal mass (9.2 cm × 6.2 cm)	BMB: (−) Esophageal mass biopsy: CD20, PAX-5, CD45, subset BCL-6, with Ki-67 proliferation rate approximately 50%–60%	Chemotherapy	Symptoms: unknown Cardiac mass: unknown Survival: no, died of status epilepticus and hypoxic respiratory failure on the 15th day of chemotherapy
10/M/Pre-B-ALL/right atrial mass/ (21)	Vomiting, body aches, shortness of breath with a episode of syncope	PR: 113 beats/min BP: 100/60 mmHg RR: 20/min	Sinus tachycardia and atrial enlargement	–	CR: enlarged cardiac silhouette TTE: a large, pedunculated, right atrial mass (12 × 18 cm), there was severely decreased antegrade flow distal to the mass, LVEF: 64%	Cardiac mass biopsy: pre-B-ALL Immunohistochemical: pre-B-ALL	Surgery + vincristine chemotherapy	Symptoms: disappeared Cardiac mass: disappeared Survival: yes, complete remission after chemotherapy
10/M/Pre-B-ALL//right atrial and right ventricle mass/ (22)	Fatigue, vomiting with a episode of syncope	–	–	–	CR: cardiomegaly TTE: a large heterogeneous right atrial mass protruding through the tricuspid valve into the right ventricle	Cardiac mass biopsy: a diffuse proliferation of medium-sized immature lymphoid cells with a high nuclear/cytoplasmic ratio, an immature chromatin pattern, and inconspicuous nucleoli Immunohistochemical: CD79a(+), CD10(+), and TdT (+); CD20(−) Flow cytometric analysis: CD 19(+), CD22(+), CD79a(+), CD10(+), and TdT (+); CD20(−)	Surgery + induction chemotherapy	Symptoms: asymptomatic after chemotherapy Cardiac mass: disappeared Survival: yes, complete remission after chemotherapy

B-ALL, B-cell acute lymphoblastic leukemia; F, female; M, male; PR, pulse rate; BP, blood pressure; RR, respiration rate; WBC, white blood cell; Hb, hemoglobin; PLT, blood platelet; CR, chest radiography; CT, computerized tomography; TTE, transthoracic echocardiography; CMR, cardiac magnetic resonance; BMB, bone marrow biopsy; IgH, immunoglobulin heavy chain; APBSCT, autologous peripheral blood stem cell transplantation.

Chest CT, TTE, cardiac magnetic resonance (CMR), and positron emission tomography-computed tomography (PET-CT) were commonly used for cardiac mass detection and pathological characteristic analysis (**Table 2**). However, the diagnosis of B-ALL largely depended on the biopsy of bone marrow and cardiac mass. The present studies showed that the positive rate for leukemic cardiac infiltration using mass biopsy and BMB was 100% (2/2) and 66.6% (4/6), respectively (**Table 2**). Notably, other than cardiac mass, kidney and esophageal invasion was occasionally observed.

After the diagnosis of B-ALL was made, the patients with severe hemodynamic disorder underwent cardiac surgery and chemotherapy, while others only adopted chemotherapy (**Table 2**). Five patients achieved cardiac mass resolution after 5 days to 2 months of chemotherapy. Five patients achieved complete remission from ALL after 2 months of chemotherapy. One patient died of respiratory failure 15 days after chemotherapy. One died of chemotherapy resistance after 25 days of treatment and one lost follow-up.

### 3.3 Myocardium Hypertrophy as the First Manifestation of ALL

T-ALL-dominant pathological characteristics are presented in **Table 3**. A young to middle-aged preference was manifested with an average age of 35 years old (ranging from 26 to 51). The left ventricular (LV) wall was most frequently involved (5/8), followed by the ventricular septum (2/8) and atrium (1/8). Progressive dyspnea was the most common symptom. Unlike the hypertensive-hypertrophy, ECG usually displayed low voltage and diffuse T-wave inversion, occasionally with ST-segment elevation (II, III, aVF) and sinus tachycardia.

**Table 3:** Myocardium hypertrophy as first manifestation of acute lymphoblastic leukemia.

Age/gender/type/first manifestation/reference	Initial symptoms/signs	Initial vital signs	Initial ECG	Initial blood test	Initial imaging examination	Definite diagnosis	Treatment regimen	Outcome
38/M/T-ALL/interventricular septum hypertrophy (23)	Vasculitic rash and horizontal diplopia	–	Diffuse T wave inversion	–	TTE: biventricular hypertrophy and speckled myocardium CMR: septal left ventricular hypertrophy (max 21 mm, normal <12 mm) with left ventricular mass; T1-weighted imaging showing two heterogeneous mass; preserved biventricular systolic function	Extraocular muscles biopsy: T-ALL	Systemic chemotherapy	Ventricular wall thickness: had normalized (10 mm) 1 month later after chemotherapy Survival: unknown
34/F/B-ALL/interventricular septum hypertrophy (24)	Vertebral pain	–	LV hypertrophy with deep, symmetrically negative T waves	–	Chest CT: enlarged abdominal lymph nodes TTE: asymmetric hypertrophy of the mid and distal septum (17 mm) with a speckled appearance of the myocardium; LVEF normal CMR: diffusely distributed myocardial hypertrophy PET-CT (18F-FDG): an intensive irregular tracer uptake in the myocardium	BMB: (−) Liquor analysis: CD19(+), CD20(+), CD79a(+) Cytogenetic analysis: t(8;14) translocation Molecular analysis: IGH rearrangement	Systemic chemotherapy	Ventricular wall thickness: had normalized 27 days later after chemotherapy Survival: no, relapsed and died
33/M/T-ALL/LV hypertrophy (25)	Progressive fatigue and dry cough	–	Sinus tachycardia and T-wave inversions	WBC: 240.9 × 10^9^/L	CR: cardiac silhouette enlargement TTE: focal left ventricular hypertrophy involving the mid- and apical segments of the anterior and anterolateral walls with mildly reduced LVEF (40%–45%); hypokinesis was noted in the hypertrophied segments. CMR: a large superior mediastinal mass and late gadolinium enhancement in the apical inferior and apical septal wall; T1-weighted imaging showing a heterogeneous mass	Peripheral blood smear: 96% blasts, T-ALL EMB: myocardial leukemia infiltration	Systemic chemotherapy	Ventricular wall thickness: had normalized of LV thickness, regional thickening, and global systolic function 1 month later after chemotherapy Survival: unknown
38/M/ALL/LV and interventricular septum hypertrophy (26)	Abdominal lymphadenopathy	–	–	–	TTE: LVEF reduce, diffuse thickening of ventricular walls CMR: diffuse inhomogeneous late gadolinium enhancement in the myocardium PET-CT (18F-FDG): high inhomogeneous uptake of 18-fluoro-deoxyglucose in the myocardium	Lymph node biopsy: ALL	Systemic chemotherapy	Ventricular wall thickness: had normalized ventricular wall thickness and systolic function after four cycles of chemotherapy Survival: unknown
51/M/B-ALL/LV and interventricular septum hypertrophy (27)	Shortness of breath on exertion	–	–	Normal	TTE: markedly thickened left ventricular wall (25 mm) and interven tricular septum, mimicking hypertrophic cardiomyopathy	EMB: atypical lymphoid cells are large, with moderately pleomorphic nuclei	–	Died 1 week after hospitalization
26/M/T-ALL/atrium and interatrial septum hypertrophy (28)	Peripheral facial paralysis and a testicular tumor	–	Sinus tachycardia, ST-segment elevation in leads II, III and aVF	WBC: 13 × 10^9^/L Hematocrit: 36% PLT: 615 × 10^9^/L	TTE: infiltrative thickening of the aortic walls, left atrium, right atrium, interatrial septum and the tricuspid annulus, and mildly depressed systolic function of both ventricles.	Axillary biopsy: T-ALL	Systemic chemotherapy	Atrium wall thickness: normalized Survival: No, relapsed and died during the fourth hospital admission
26/M/T-ALL/LV hypertrophy (29)	Progressive dyspnea on exertion and low-grade fever	–	Right axis deviation	–	CR: cardiomegaly Radionuclide ventriculography: LVEF 22% TTE: LVEF 40%, left ventricular hypertrophy (23 mm) and a large left atrial mass PET-CT (18F-FDG): abnormal hypermetabolic lesions involving the left atrium cavity and the myocardium	BMB: (−) EMB: T-ALL, atypical lymphoid cells with irregular nuclear membrane and hyperchromasia Immunohistochemistry: CD3 positivity in the atypical lymphoid cells	Systemic chemotherapy	Ventricular wall thickness: had normalized after 2 cycles of chemotherapy Survival: No, relapsed and died 6 months later
40/M/T-ALL/LV hypertrophy (30)	Progressive dyspnea	–	Low voltage and diffuse T-wave inversions	–	CR: cardiomegaly and pulmonary congestion with right pleural effusion TTE: LVEF 12.2%, echocardiographic diastolic dysfunction was grades 3–4 with left atrial enlargement (E/A 2.86, deceleration time 83 ms, e’ 0.030 m/s, E/e’ 25.13, LA diameter 53 mm, LA volume index 60.5 ml/m^2^). CMR: biventricular hypertrophy with diffuse late gadolinium enhancement	BMB: (−) EMB: atypical lymphocytic infiltration in myocardium	Systemic chemotherapy	Ventricular wall thickness: normalized after chemotherapy LVEF: recovered from 12.2% to 63.0% after chemotherapy Survival: unknown

T-ALL, T-cell acute lymphoblastic leukemia; B-ALL, B-cell acute lymphoblastic leukemia; LV, left ventricular; F, female; M, male; WBC, white blood cell; PLT, blood platelet; CR, chest radiography; CT, computerized tomography; TTE, transthoracic echocardiography; BMB, bone marrow biopsy; EMB, endomyocardial biopsy; CMR, cardiac magnetic resonance; LVEF, left ventricular ejection fraction.

The leukemic infiltrated myocardium was characterized by speckle, hypokinesis, and/or multifocal regional thickening. TTE had an advantage in accessing the infiltration location, myocardial kinetics, and systolic function. CMR helped in recognizing the neoplasia myocardium infiltration by showing diffuse inhomogeneous late gadolinium enhancement, while PET-CT could monitor the relapse of ALL infiltration with manifestation of intensive irregular tracer uptake in the myocardium.

The diagnosis of ALL myocardial infiltration largely depended on the endomyocardial biopsy (EMB) rather than BMB. The present studies (**Table 3**) showed that the positive rate for EMB and BMB was 100% (4/4) and 0 (0/3), respectively. Occasionally, myocardium infiltration was accompanied by extraocular muscles and lymph node invasion.

After diagnosis of ALL with myocardium infiltration, all patients adopted systemic chemotherapy (**Table 3**) except for a 55-year-old male patient. Myocardium hypertrophy had gradually vanished in all patients who received chemotherapy. The one who did not accept chemotherapy died of deteriorated conditions 1 week after diagnosis. Three patients were relapsed and disseminated during follow-up and died after 6–14 months of treatment. However, the long-term outcomes of the other four patients were unknown.

### 3.4 AMI as the First Manifestation of ALL

A total of six ALL cases reported AMI-like symptoms as the first clinical manifestation (**Table 4**), with an average age of 42 years old (ranging from 24 to 61) and exception of a 2-year-old boy. Surprisingly, 66.7% (4/6) of patients showed severe chest pain but without significant coronary artery obstruction, whereas 33.3% (2/6) of patients with severe coronary artery stenosis showed absence of chest pain. Initial ECG showed that 75% (3/4) of patients presented ST-segment elevation, and 25% (1/4) of patients presented T-wave inversion. Q wave had never been observed in any patient, although cTnI was significantly elevated in all patients with a range of 0.2–1.19 ng/ml (7–10-fold increase).

**Table 4:** Acute myocardial infarction as first manifestation of acute lymphoblastic leukemia.

Age/gender/type/first manifestation/reference	Initial symptoms/signs	Initial ECG	Initial blood test	Initial imaging examination	Definite diagnosis	Treatment regimen	Outcome
61/F/B-ALL/STEMI/ (31)	Severe chest pain	ST-segment elevation	cTnT: 1.19 ng/ml (normal <0.1 ng/ml)	Coronary angiography: no significant stenosis TTE: severe hypokinesis of the anterior segment and a dense thick mass in the right atrioventricular groove and pericardium	Bone marrow biopsy: (−) Peripheral blood smear: (−) EMB: Pre-B-ALL, diffuse infiltration of leukemic cells	Chemotherapy	ST-segment: the broad anterior ST-segment elevation gradually returned to baseline Survival: yes, complete remission
39/M/T-ALL/NSTEMI/ (32)	Dull chest pain	T-wave inversion without ST-segment elevation	cTnT: 0.2 ng/ml (normal <0.03 ng/ml)	CCTA: abnormal soft tissue in the left atrioventricular groove resulting in approximately 50% stenosis of the left circumflex artery TTE: marked asymmetric thickening of the lateral LV wall CMR: marked asymmetric thickening of the lateral LV wall	BMB: small lymphoblasts comprising approximately 50% of total cellularity; T-ALL	Chemotherapy	T-wave: T-wave inversion gradually returned to baseline Survival: yes, complete remission
24/M/ALL/STEMI/ (33)	Severe chest pain, dyspnea, and diaphoresis	ST-segment elevation	–	Coronary angiography: no significant stenosis TTE: severe pericardial effusion, lateral akinesis with global LVEF preserved CMR: confirmed the direct infiltration of the neoplasia in the pericardium and myocardium	Autopsy: leukemia myocardium infiltration	Anti-ischemic and opiate analgesic drugs	Died a few days later by respiratory failure
34/M/T-ALL/STEMI/ (34)	Severe chest pain and dyspnea	ST-segment elevation	–	TTE: hypertrophy in right ventricular outflow tract as well as interventricular septum and posterior wall, LVEF were normal CMR: tumoral infiltration of septum and posterior wall and right ventricular free wall	Cytological (pericardial effusion): atypical lymphoid cells	Chemotherapy	ST-segment: resolution of ST segment elevation in right precordial derivations
52/M/T-ALL/AMI/ (35)	Asymptomatic	–	–	PET-CT: a cavitary lesion in the upper lobe of the right lung	Autopsy (macroscopic): the lumina showed stenoses of 70% (right coronary artery) to 95% (left anterior descending and left circumflex coronary arteries), with no thrombosis identified Autopsy (microscopic): 3 major coronary artery branches were heavily infiltrated with leukemic cells Immunophenotyping: T-ALL, CD2(+), CD3(+), CD7(+), CD4(+), CD8(+), CD34(−), TdT(−), CD1a(-), CD20(−)	–	Died of hypovolemic shock and acute respiratory failure
2/M/ALL/AMI/ (36)	Asymptomatic	–	–	An abdominal ultrasound: an enlarged liver with an abnormal round nodule, 2 cm in diameter, in the left lobe. TTE: LVEF (35%), abnormal atrial and ventricular wall motion and a pericardial effusion of 10 mm thickness around the heart.	Autopsy (macroscopic): the left main coronary artery was markedly dilated and contained a thrombus Autopsy (microscopic): leukemic infiltration compressing the left coronary artery with thrombotic occlusion of the vessel lumen	Digitalis and furosemide	Died of cardiac arrest

T-ALL, T-cell acute lymphoblastic leukemia; B-ALL, B-cell acute lymphoblastic leukemia; STEMI, ST-segment elevation myocardial infarction; AMI, acute myocardial infarction; LV, left ventricular; CCTA, coronary computed tomography angiogram; F, female; M, male; WBC, white blood cell; PLT, blood platelet; CR, chest radiography; CT, computerized tomography; TTE, transthoracic echocardiography; BMB, bone marrow biopsy; EMB, endomyocardial biopsy; CMR, cardiac magnetic resonance; LVEF, left ventricular ejection fraction.

Coronary imaging examinations, including coronary angiography and coronary computed tomography angiogram, showed that 3/6 of patients presented normal coronary artery, 1/6 of patients presented moderate stenosis (50%) in the left circumflex artery, and 2/6 of patients presented severe stenosis (70%–95%) in the right coronary artery, left anterior descending artery, left circumflex artery, or left main coronary artery. The stenosis of coronary artery caused by leukemia infiltrating was centripetal. Unlike conventional AMI, there were no plaques (i.e., atherosclerotic and calcified plaque) in the coronary artery. The lumen stenosis and occlusion were secondary to the vascular wall thickening caused by leukemia infiltration and the acute leukemia clots. Interestingly, ALL patients with AMI manifestation usually had asymmetric myocardial hypertrophy and/or cardiac mass, indicating the leukemic invasion in both coronary artery and myocardium (for details, see **Table 4**).

In these patients, 50% (3/6) did not show stenosis or occlusion of any coronary artery. Therefore, stent and thrombolytic therapy were not applied. Instead, two patients adopted chemotherapy and achieved a complete normalization of ECG (ST segment and T wave). However, the patient who only adopted anti-ischemic and opiate analgesic therapy died of respiratory failure. In patients who did not accept coronary angiography or CCTA (3/6), one patient adopted chemotherapy and achieved complete regression of ST segment and one patient only accepted symptomatic treatment and died of cardiac arrest. The other one without any medical treatment died of hypovolemic shock and acute respiratory failure. These outcomes indicate that chemotherapy is adequate for coronary leukemic invasion ALL patients.

## 4 Discussion

In some cases, cardiac manifestation could be the first clinical finding of ALL. These patients may first visit cardiologists instead of oncologists. Notably, the blood test could be completely normal at the onset of cardiac symptoms. This usually led to clinical tragedy due to misdiagnosis or delayed diagnosis. Although few cases were reported in the literature, many patients may have been misdiagnosed as heart disease and died. It is presumable that in the real world, the morbidity of ALL patients with heart disease as the primary manifestation may be much higher than we think. Therefore, it is helpful to document the clinical features of these patients and put forward feasible methods to avoid misdiagnosis and mistreatment.

### 4.1 Cardiac Manifestations Precede ALL Diagnosis

In the included 30 cases, 80% (24/30) of patients presented cardiac manifestations before ALL was diagnosed. In total, 33.3% (8/24) of cases (7–14) had detected a persistent pericardial effusion and/or cardiac tamponade, 33.3% (8/24) of cases (15–22) had detected a cardiac mass, and 33.3% (8/24) of cases (23–30) had detected severe cardiac hypertrophy by TTE (7–18, 20–22, 24–30) and/or CMR (19, 23) test. All of these 24 cases were diagnosed with ALL by further tests of BMB (7, 16–19), BMA (8–11, 14), EMB (25, 27, 29, 30), cytologic analysis (12, 13, 15, 24), or tissue biopsy (20–23, 26, 28).

However, 20% (6/30) of patients presented severe chest pain (31–34) or silent (asymptomatic) myocardium ischemia (35, 36) as the first manifestation of acute leukemia relapse, in which one patient (32) manifested an abnormal soft tissue in the left atrioventricular groove with a 50% stenosis in the left circumflex artery by CCTA; one patient (35) showed heavy leukemic infiltration in three major coronary artery branches at autopsy (microscopic); and one patient (36) showed leukemic infiltration with thrombotic occlusion of the vessel lumen at autopsy (microscopic). These results indicated that the AMI-like clinical manifestations (32, 35, 36) were possibly a result of coronary artery leukemic infiltration or leukemic thrombus.

### 4.2 Experience and Lessons Learned From Clinical Misdiagnosis

#### 4.2.1 Experience

In ALL patients with massive pericardial effusion or cardiac tamponade, pericardiocentesis is an emergency measure to relieve cardiac tamponade symptoms and detect the malignant hematological cells (up to 50% positive rate). Therefore, it should be immediately conducted for diagnosis and treatment purposes once confirmed by echocardiography, especially for young people without previous cardiac problems or autoimmune diseases. In addition, the patients presented massive pericardial effusion or cardiac tamponade as the first sign of T-ALL usually manifested bleeding tendency due to the abnormal blood elements (8, 12). This needs to be considered before pericardiocentesis.

In ALL patients with intracardiac mass, the symptoms largely depend on the mass dimension and location. A large mass that occupied the entire cardiac chamber could cause severe hemodynamic disorder, such as syncope (21, 22). A right heart mass could cause systemic congestion, such as peripheral edema and ascites (19). In contrast, a LV medium-size mass could cause acute pulmonary congestion and present progressive dyspnea (18, 20). Moreover, a LV apex hypermobile mass could cause outflow tract obstruction and thus reduce cardiac output, leading to insufficient coronary perfusion and angina (17).

In ALL patients with infiltration-induced hypertrophy, the systolic function could be reduced depending on the region and severity of infiltration. The LV free wall infiltration could cause a reduced left ventricular ejection fraction (LVEF) (25–30) with progressive dyspnea, while the ventricular septum infiltration could have normal LVEF (23, 24) without cardiac symptoms.

In ALL patients with AMI, the severity of cardiac symptoms did not match the degrees of coronary stenosis. The patients who presented abnormal ECG (ST-segment elevation or T-wave inversion) and significantly elevated cTnI (0.2–1.19 ng/ml) experienced acute, severe chest pain with absence of coronary stenosis (31–34). The possible explanations include: (1) the ventricular infiltration of leukemic cells caused coronary artery compression; (2) vascular wall leukemic infiltration-induced centripetal thickening; (3) coronary microvessel occlusion; (4) coronary artery spasm and constriction; and (5) diffuse myocardial injury.

In contrast, two patients with severe coronary stenosis or occlusion were asymptomatic, including a case with occluded left main coronary artery (36) and a case with multiple severe coronary stenoses (35). The absence of symptoms in the conventional AMI was usually observed in elderly patients and patients with diabetics due to the insensitivity of the neve system. However, these two patients were young and without diabetics. Whether these patients had nerve injuries was unclear.

Taken together, ALL patients with severe chest pain and AMI-like ECG change and cTnI elevation may not be the actual onset of AMI caused by the rupture of coronary plaques. Instead, these changes could derive from the leukemic infiltration. Chemotherapy, other than coronary intervention, is usually effective. However, coronary angiography is required to identify the silent AMI. There was no knowledge about the pathologic feature of the coronary thrombus in the AMI-like ALL patients (e.g., the leukemic cluster or thrombus caused by the plaque rupture). However, this is critical for determining the proper coronary intervention strategy. Although currently not available, the autopsy data will address this issue.

#### 4.2.2 Lessons

##### 4.2.2.1 In ALL Patients With Cardiac Tamponade as the First Manifestation

A 7-year-old girl (10) presented cardiac tamponade as the first sign of T-ALL. She had a negative result in peripheral blood smear and pericardiocentesis. Therefore, she did not accomplish the BMB test due to these negative results. Her cardiac symptoms were improved after symptomatic treatment (broad-spectrum antibiotics, prednisone, lasix, digoxin). Unfortunately, she was diagnosed as ALL-L2 by BMB at her second hospital visit (1 month later) and died 1 year after the diagnosis.

In contrast, an 18-year-old female patient (7) presented cardiac tamponade as the first sign of T-ALL also showed negative results in peripheral blood smear and pericardiocentesis. However, she had BMB test due to a large mediastinal mass indicated by TTE and swollen mediastinal lymph nodes indicated by chest CT. She was diagnosed with T-ALL by BMB and achieved complete remission after chemotherapy. Moreover, a 15-year-old male patient (16) presented cardiac mass as the first sign. He had accomplished a BMB test due to fever, hepatosplenomegaly, and abnormal blood test (WBC: 63 × 10^9^/L, Hb: 57 g/L, PLT: 10 × 10^9^/L). He obtained a definite diagnosis of B-ALL by BMB and achieved complete remission after chemotherapy.

These cases indicated that the BMB is critical for the diagnosis of ALL. It should be routinely conducted in patients with cardiac mass or massive pericardial effusion or cardiac tamponade for unknown reason, especially for young people, even the peripheral blood test and pericardiocentesis results are normal.

Although BMB was important for ALL diagnoses, BMB was sometimes negligently conducted. For instance, a 13-year-old male patient (17) presented a left ventricular mass as the first sign of B-ALL was diagnosed as hypereosinophilic syndrome due to the careless examination of bone marrow. In fact, there was at least 40% lymphoblast in his bone marrow section, which was found 3 months later by retrograde reviewing the bone marrow section. Unfortunately, this misdiagnosis largely delayed his treatment and caused his death. A similar misdiagnosis was made in another 13-year-old male patient (37). Therefore, BMB needs to be routinely and carefully reviewed in the highly suspected ALL patients.

### 4.3 In ALL Patients With Myocardium Hypertrophy as the First Manifestation

A 26-year-old male patient (29) had accomplished EMB due to left ventricular hypertrophy and large left atrial mass. He obtained a definite diagnosis of T-ALL by EMB and had a complete resolution of hypertrophic myocardium and left atrial mass after chemotherapy. By contrast, a 40-year-old male patient (30) had accomplished EMB due to low voltage in the ECG with remarkable left ventricular hypertrophy. He obtained a definite diagnosis of T-ALL by EMB and had a complete resolution of myocardium hypertrophy after chemotherapy. In addition, a 33-year-old male patient (25) had accomplished EMB due to delayed gadolinium enhancement in the apical inferior and apical septal wall and a large superior mediastinal mass. He was diagnosed with T-ALL by EMB and reached a complete resolution for the myocardium hypertrophy and superior mediastinal mass after chemotherapy.

### 4.4 In ALL Patients With Clinical Manifestations of AMI

A 39-year-old male (32) presented severe chest pain with T wave inversion. CCTA showed extensive abnormal soft tissue in the left atrioventricular groove, right atrium and lateral pericardium, resulting in about 50% stenosis of the left circumflex artery. It indicated that the stenosis was likely caused by neoplasia myocardium infiltration. Notably, his cTnI level was slightly increased (0.2 ng/ml, reference range <0.03). Considering only a 50% stenosis in the coronary artery, the elevation of troponin I was possibly caused by a neoplasia myocardium infiltration rather than coronary stenosis. He had accomplished BMB due to asymmetric left ventricular hypertrophy and delayed gadolinium enhancement in the left ventricular lateral wall. He obtained a definite diagnosis by BMB and achieved a remarkable resolution of left ventricular hypertrophy with normalized T wave after five months of chemotherapy.

In contrast, the coronary angiography of a 61-year-old female patient (31) showed no stenosis. Interestingly, the cTnI levels (1.19 ng/ml) were significantly elevated, and the imaging examinations (TTE, enhanced CT, and gallium scintigraphy) revealed an abnormal neoplasia infiltration of the myocardium and pericardium. Therefore, the chest pain was likely caused by neoplasia cardiac infiltration rather than coronary stenosis. She had accomplished EMB examination, although the result of BMB test was negative. She obtained a definite diagnosis of B-ALL by EMB and had a remarked resolution of cardiac infiltration after two weeks of chemotherapy.

The pattern of changes in high-sensitivity troponin I (hs-TnI) could be helpful to distinguish AMI-like leukemia from the conventional AMI. For instance, in the conventional AMI, the TnI curve contains ascending and descending phases (with a peak) due to acute death of a large numbers of cardiomyocytes. Owing to the one-time cardiac death, the curve will manifest a clear peak 10–24 h after the onset. In addition, conventional AMI patients have no evidence of neoplasia myocardium infiltration. In contrast, the AMI-like ALL presented neoplasia myocardium infiltration (31–34) and had no evidence of severe coronary stenosis (31, 33, 34). The TnI curve could show an ascending trend and then stabilization at a higher level without a typical peak due to the continuous cardiomyocyte injury caused by leukemic cell infiltration. EMB needs to be conducted for ALL diagnose, but it should not be conducted in conventional AMI patients due to the risk of ventricular rupture. Unfortunately, all of the AMI-like ALL patients (31–36) in the present study did not monitor the dynamic changes of cTnI levels and thus missed the opportunity to understand the underlying pathology.

Notably, two B-ALL patients (17, 18) presented cardiac mass as the first sign with remarkably elevated eosinophils in peripheral blood. In fact, in some cases, eosinophilia might be the initial presentation of B-ALL (38–40). As a large number of eosinophils might mask underlying or coexisting leukemia due to the absence of lymphoblasts in peripheral blood (37), the BMB is strongly recommended in patients presented cardiac mass with eosinophilia. Furthermore, Sumners et al. (2) compared 1-week and 1-month antemortem peripheral leukocyte count and found that the numbers of the peripheral leukocytes were significantly higher in ALL patients with cardiac infiltration than those without cardiac infiltration. Thus, the increased levels of eosinophil (17, 18, 37), leukocyte (8, 11, 16–19, 25, 28), and lymphoblast (8, 9, 11, 19, 25) might be a clue of ALL, and the blood test and peripheral blood smear should be routinely conducted in patients with massive pericardial effusion or cardiac tamponade, cardiac mass, or suspected myocardium infiltration. In rare cases, the laboratory examinations, including pericardial effusion cytologic examination, routine blood test, peripheral blood smear, BMB, and EMB showed negative results. If highly suspected, the flow cytometric measurement, immunophenotypic, immunohistochemical, cytogenetic, and genome-wide single nucleotide polymorphism study should be performed to search T/B cell markers.

For instance, Fournier et al. (41) claimed a specific L3-IgH rearrangement in B-ALL with eosinophilia. Consistent with this, the rearrangement of L3-IgH was observed in a patient (37) with eosinophilia who was finally diagnosed as B-ALL. Therefore, the L3-IgH rearrangement test is helpful for screening ALL patients who presented cardiac mass with eosinophilia.

Importantly, cardiac symptoms or signs could be absent in the AMI-like ALL patients. For example, a 52-year-old male patient (35) with a history of untreated T-ALL, the PET-CT indicated a pulmonary lesion, he did not conduct ECG and cTnI test due to the absence of cardiac symptoms, a few days later, he died of hypovolemic shock and acute respiratory failure. His autopsy revealed multiple, severe coronary stenosis (70% in right coronary artery, 95% in left anterior descending and 95% in left circumflex artery), indicating that he likely experienced a silent (asymptomatic) myocardium ischemia before death (the cTnI level has not been documented). Similarly, a 2-year-old boy (36) had a complete remission of ALL after chemotherapy. However, during the follow-up, the auxiliary examinations (WBC: 0.6 × 10^9^/L, liver nodule, splenomegaly, pericardial effusion) indicated a relapse of ALL. He did not accomplish ECG and cTnI examination due to the absence of cardiac symptoms. Unfortunately, he died of cardiac arrest 17 days later. His autopsy revealed massive leukemic cell infiltration in the left main coronary artery with a thrombus occlusion of the vessel lumen. This indicates that in the patients with a deteriorated or disseminated ALL, regardless of cardiac symptoms, ECG and cTnI should be routinely examined. Once the abnormality was observed, coronary angiography or CCTA should be conducted.

### 4.5 Experience and Lessons Learned From Clinical Therapy

In ALL patients with massive pericardial effusion or cardiac tamponade presented, mild clinical condition usually achieved complete remission with a resolution of pericardial effusion after chemotherapy (7, 9, 12, 14). However, a 27-year-old male patient (8) presented severe condition with developed bilateral parietal lobe hemorrhage with reaccumulated pericardial effusion after 4 days of chemotherapy and died of cardiorespiratory arrest 5 days later. Also, a 7-year-old girl (10) who presented severe clinical condition did not obtain remission after 4 cycles of chemotherapy and finally developed systemic and cerebral diffusion and died after 1 year of treatment. These indicate that the outcome of chemotherapy for the ALL patients presented massive pericardial effusion or cardiac tamponade largely depends on the severity of the basic condition of ALL itself. The cardiac effusion would usually be absorbed after chemotherapy, and cardiac dysfunction has not been observed.

Interestingly, a 15-year-old female patient (11) with persistent pericardial effusion developed a mediastinal mass and two large right atrium masses with severe chest pain after 1 week of chemotherapy. She was lucky since the histologic sections revealed necrotic thrombus for the right atrial masses and chronic mediastinitis for the mediastinal mass. Thus, her chemotherapy continued and achieved complete remission with a resolution of pericardial effusion 1.5 years later. Whether the necrotic thrombus and mediastinitis were associated with the chemotherapy remains unclear, although very likely.

The ALL patients with myocardium hypertrophy or cardiac mass, if manifested with normal or preserved LVEF, usually tolerate chemotherapy well. Most of them could achieve a complete resolution of myocardium hypertrophy and mass. However, a 26-year-old male patient (29) presented significantly decreased LV systolic function (LVEF 22%) and developed chemotherapy resistance on the fourth treatment cycle. He died of ALL relapse after 6 cycles of chemotherapy. In contrast, a 40-year-old male patient (30) presented remarkably reduced LV systolic function (LVEF 12.2%) with no evidence of chemotherapy resistance completed the chemotherapy and achieved a resolution of LV hypertrophy with a complete recovery of LV systolic function (LVEF 63.0%) at the end of chemotherapy. These indicated that no matter the patients with normal or reduced LVEF, chemotherapy needs to be conducted, and the outcome largely depends on the sensitivity of the patients to the chemotherapy.

ALL patients could present severe chest pain, ischemic ECG changes (ST-segment elevation and/or T-wave inversion) and significant elevation of cTnI (7–10-fold increase), a similar manifestation of AMI (31–34). However, coronary angiography or CCTA did not show significant coronary stenosis (31–33). Therefore, the ECG changes and elevated cTnI levels were likely caused by neoplasia myocardium infiltration, other than the coronary ischemic myocardial injury. This was proved by the success of chemotherapy, with achievement of symptom relief and normalization of ECG, although the curvilinear trajectory of cTnI had not been documented.

### 4.6 General Perspectives of Anticancer Therapy

The tolerance of ALL patients with cardiac infiltration is likely a concern for anticancer therapy, especially in consideration of cardiac toxicity. However, the cardiac manifestation in ALL patients is primarily caused by neoplasia infiltration rather than the primary cardiac problem. Therefore, anticancer therapy can generally relieve and gradually vanish cardiac symptoms. In fact, in patients who accepted chemotherapy, a complete resolution was achieved in 62.5% (5/8) of patients with pericardial effusion or cardiac mass (**Tables 1**, **2**), and 100% of patients with myocardium hypertrophy (7/7) and AMI-like cardiac injury (3/3) (**Tables 3**, **4**). Notably, deterioration of clinical condition with chemotherapy was also detected in 25% (2/8) of patients with cardiac tamponade or cardiac mass (**Tables 1**, **2**). Considering that these patients had a severe basic condition before chemotherapy, it indicates that the patients with severe clinical condition may not tolerate chemotherapy well. Therefore, chemotherapy needs to be evaluated cautiously and adjusted individually before conducting, such as the time point, course of treatment and drug selection (avoiding drugs with severe cardiac toxicity). Moreover, 42.9% (3/7) of ALL patients with myocardium hypertrophy relapsed after chemotherapy (**Table 3**), although the myocardial infiltration itself was highly sensitive to chemotherapy, indicating a poor prognosis for these patients.

Considering leukemia prone to recurrence, TTE, routine blood test, peripheral blood smear, cTnI, and ECG should be conducted to monitor the relapse during the follow-up phase.

## 5 Management Recommendations

We put forward suggestive management procedures for the diagnosis and treatment of the ALL patients who initially presented as cardiac disease:

1. In young patients who present triad of pericardial effusion, systemic congestion and/or hypotension, TTE should be performed to detect pericardial effusion or intracardiac mass. Pericardiocentesis is recommended in patients with cardiac tamponade for symptom relief and etiological judgment. However, digitalis therapy is not recommended in patients with cardiac tamponade due to nonoptimal response. BMB is strongly recommended to rule out ALL in patients with persistent pericardial effusion or tamponade without basic heart disease.
2. For patients with myocardium hypertrophy but ECG showed low voltage and diffuse T-wave inversion, CMR and/or PET-CT are strongly recommended to identify the possible myocardium infiltration, especially for patients with asymmetric ventricular hypertrophy without hypertension. If suspected with myocardium infiltration, EMB should be conducted.
3. For young patients with AMI-like clinical manifestations, CCTA and/or coronary angiography need to be performed to identify the coronary stenosis, and the dynamic changes of TnI levels and ECG also need to be monitored. The conventional AMI usually has a TnI peak and typical ST and T-wave regression with or without Q-wave formation. However, neoplasia myocardium infiltration-induced AMI-like manifestations usually come with a stabilized TnI at a high level and consistent ECG changes without Q-wave formation. Once AMI is excluded, BMB and EMB need to be conducted to rule out or confirm the diagnosis of ALL.
4. Tissue biopsy is recommended in patients with cardiac mass or suspected neoplasia infiltration. If BMB and EMB showed negative results, the flow cytometric measurement should be performed to search T/B cell markers if highly suspected. L3-IgH rearrangement examination by sequencing is recommended in patients with eosinophilia since it could be a B-ALL marker.
5. Once the definite diagnosis is made, systemic chemotherapy should be carried out as soon as possible in ALL patients who presented massive pericardial effusion or cardiac tamponade, cardiac mass, myocardium hypertrophy and/or AMI-like myocardium injury. LV dysfunction is not an absolute contraindication to chemotherapy. In most cases, the cardiac function could be significantly improved after chemotherapy because the cardiac dysfunction is caused by neoplasia infiltration rather than the primary heart disease. However, chemotherapy should be adopted with careful evaluation and individualized in patients with severe basic conditions.
6. For patients with complete remission, TTE, cTnI, ECG, routine blood test and peripheral blood smear should be routinely conducted to monitor the relapse during follow-up.

**Figure 1:**
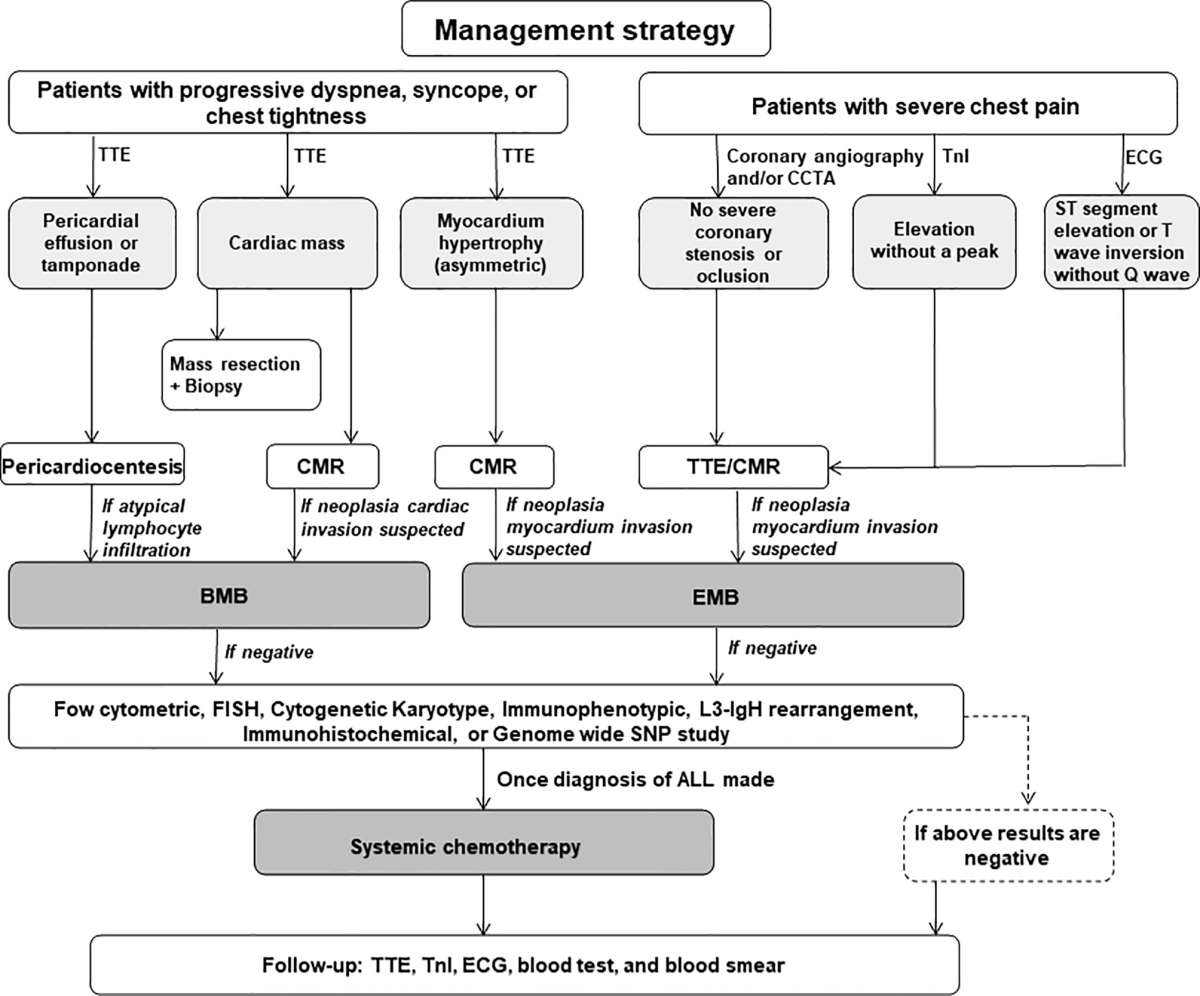
The management recommendations for the acute lymphoblastic leukemia patients with cardiac problems as the first manifestation. TTE, transthoracic echocardiography; CCTA, coronary computed tomography angiogram; TnI, troponin I; CMR, cardiac magnetic resonance; EMB, endomyocardium biopsy; FISH, fluorescence *in situ* hybridization; SNP, single nucleotide polymorphism; ALL, acute lymphoblastic leukemia.

These recommendations are illustrated in **Figure 1**.

## Data Availability Statement

The original contributions presented in the study are included in the article/supplementary material. Further inquiries can be directed to the corresponding author.

## Author Contributions

YW and ZL conceived and designed this study as well as drafted the manuscript. ZL and JC carried out the literature search and collected and organized data. YW was responsible for data interpretation, revision, and approval of the manuscript. All authors contributed to the article and approved the submitted version.

## Funding

This work was supported by grants awarded to YW from the National Natural Science Foundation of China (NSFC, Grant Nos. 81270304, 81873507, and 81420108004).

## Conflict of Interest

The authors declare that the research was conducted in the absence of any commercial or financial relationships that could be construed as a potential conflict of interest.

## Publisher’s Note

All claims expressed in this article are solely those of the authors and do not necessarily represent those of their affiliated organizations, or those of the publisher, the editors and the reviewers. Any product that may be evaluated in this article, or claim that may be made by its manufacturer, is not guaranteed or endorsed by the publisher.
